# Characterization of Novel Fragment Antibodies Against TNF-alpha Isolated Using Phage Display Technique

**DOI:** 10.22037/ijpr.2019.1100646

**Published:** 2019

**Authors:** Ali Akbar Alizadeha, Maryam Hamzeh-Mivehroud, Elnaz Haddad, Nazanin Haddad, Mehdi Sharifi, Samin Mohammadi, Samira Pourtaghi-anvarian, Siavoush Dastmalchi

**Affiliations:** a *Biotechnology Research Center, Tabriz University of Medical Sciences, Tabriz, Iran.*; b *School of Pharmacy, Tabriz University of Medical Sciences, Tabriz, Iran.*; c *Student Research Committee, Tabriz University of Medical Sciences, Tabriz, Iran.*; d *Faculty of Pharmacy, Near East University, POBOX: 99138, Nicosia, North Cyprus, Mersin 10, Turkey.*; 1 A. A. A. and M. H. M. contributed equally to this work.

**Keywords:** TNF-α, Domain antibodies, Recombinant protein production, Molecular docking, Phage display

## Abstract

Tumor necrosis factor alpha (TNF-α) is an inflammatory cytokine which plays crucial roles in pathogenesis of inflammatory diseases. The current study aimed to investigate the binding abilities of I44 and I49 domain antibodies to TNF-α. The dAbs were expressed in bacterial expression system and purified by affinity chromatography using Ni-sepharose column. The expression and purity of the proteins were evaluated using western blotting and SDS-PAGE techniques, respectively. ELISA experiment showed that I44 and I49 dAbs bind to TNF-α with the binding constants (K_d_) of 5.18 ± 1.41 and 2.42 ± 0.55 µM, respectively. The inhibitory effect of dAbs on TNF-α biological effect was determined in MTT assay in which I44 and I49 prevented TNF-α cell cytotoxicity with IC_50_ values of 6.61 and 3.64 µM, respectively. The identified anti-TNF-α dAbs could bind to and inhibit TNF-α activity. The dAbs activities can be attributed to their ability to establish hydrogen bonds as well as hydrophobic contacts with TNF-α. The results of the current study can pave the way for further structural studies in order to introduce new more potent anti-TNF-α antibodies.

## Introduction

Cytokines, as a result of inflammation, lie at the heart of chronic inflammatory and autoimmune diseases. Tumor necrosis factor alpha (TNF-α) is one of the crucial cytokines, as it directly initiates the inflammatory processes and also activates the other inflammatory cytokines (1). Although the existence of a tumor necrosis inducing mediator had been revealed more than a century ago (2), TNF-α was first identified by Carswell *et al.* in 1975, as an endotoxin-induced serum factor responsible for the necrosis of the turmeric cells (3). TNF-α is produced as a 26 kDa cell anchored protein, mainly by activated monocytes and macrophages, and subsequently undergoes enzymatic degredation by TNF-α converting enzyme (TACE) leading to a soluble 17 kDa mature protein (2, 4). At the physiological levels, TNF-α is involved in maintaining homeostasis by regulating the body′s circadian rhythm as well as participation in immunity responses, embryonic development, and sleep regulation (1, 5 and 6). Rheumatoid arthritis (RA) and Crohn′s disease (CD) are two well-known examples of inflammatory diseases in which the prominent role of TNF-α has been proved (7, 8). Beside of various kinds of inflammatory disorders caused by unregulated production of TNF-α, it has been demonstrated that the increased TNF-α serum levels may augment manic and depressive episodes in bipolar disorders (9). Due to crucial role of pathological levels of TNF-α in different inflammatory complexities, this key cytokine has attracted much attraction as a suitable target for pharmacotherapy of inflammatory diseases. Among different strategies of TNF-α inhibition, recent studies have focused on the use of antibodies to treat patients stricken by high levels of TNF-α (10, 11). Therefore, most of the therapeutics in the market used for TNF-α inhibition are based on antibodies such as Infliximab (Remicade)®, adalimumab (Humira)®, golimumab (Simponi)®, etanercept (Enbrel)®, and certolizumabpegol (CIMZIA)® (12-16). Although these anti-TNF-α therapeutics are approved for treatment of inflammatory conditions due to their high specificity, the problems regarding their high production cost, immunogenicity, low clearance rate and stability have decreased their popularity in the pharmaceutical market (8, 17-19). For that reason, developing new anti-TNF-α inhibitors seems to be essential from pharmacokinetics, efficacy, and cost points of view (20). Antibody fragments are suitable candidates for targeting TNF-α, as their smaller size provides better pharmacokinetics properties while the degree of specificity remains intact (21, 22). In our previous study, two phage particles displaying dAbs against TNF-α was identified using phage display technology (23). The purposes of the current work were to express and purify these anti-TNF-α dAbs (*i.e.*, I44 and I49) in order to investigate their binding ability to TNF-α and assess their ability to inhibit TNF-α biological activity. To gain insight into the mode of interactions of the identified anti-TNF-α dAbs with TNF-α, molecular docking study was performed. 

## Experimental

*Reagents*

Tryptone, yeast extract, isopropyl-D-thio galactopyranoside (IPTG), Triton X-100, potassium acetate, phenylmethylsulfonyl fluoride (PMSF), and N,N,N′,N′-tetra methyl ethylene diamine (TEMED), were purchased from AppliChem (Darmstadt, Germany). 3,3′,5,5′-Tetramethylbenzidine (TMB),Acrylamide, N,N′-methylene-bis-acrylamide, and thiazolyl blue tetrazolium bromide (MTT) were obtained from Sigma Aldrich, USA. Mouse anti-His primary antibody was prepared from GE Healthcare (Sweden). Goat anti-mouse IgG-HRP secondary antibody was purchased from Santa Cruz Biotechnology (USA). BM Chemiluminescence Western Blotting kit was purchased from Roche Diagnostics GmbH (Mannheim, Germany). Adalimumab was a kind gift from CinnaGen Company, Iran. All chemicals and reagents were of molecular biology grade. Ultra pure water, produced by Milli-Q system (Millipore Corporation, Bradford, MA, USA), was used for preparation of all solutions. 

*Expression and purification of anti –TNF-α domain antibodies*

Tomlinson I and J scfv libraries were used for identification of specific anti-TNF antibodies as described in our previous study in which phage harboring I44 and I49 dAbs demonstrated high affinity towards TNF-α (23). 

In order to express I44 and I49 proteins, *E. coli origami* *(DE3) *was infected by phage particles harboring I44 and I49 coding sequences. To do this, from each serially diluted phage, 10 µL was added to 100 µL *E. coli origami* *(DE3) *at OD 0.4 followed by incubation at 37 °C for 30 min without shaking. Then the mixtures were poured onto LB plates supplemented with 100 µg/mL ampicillin and 1% glucose. In the next day, a single colony of *E. coli origami (DE3),* infected with phage, was inoculated into 10 mL LB-ampicillin medium and cultured overnight at 37 °C. The overnight culture was diluted 1:50 in 500 mL LB medium and incubated at 37 °C while shaking. At OD of 0.9, IPTG with final concentration of 1 mM, was added and the temperature was set to 30 °C for overnight incubation. Then the culture was centrifuged at 3,000×g for 15 min and the harvested bacterial pellet was resuspended in lysis buffer (Tris 50 mM pH 8, NaCl 150 mM, Triton 1%, lysozyme 0.1 mg/mL, DNAse 10 µg/mL, β-mercaptoethanol 0.1%, PMSF 1.4 mM). The suspension was freeze-thawed three times using liquid nitrogen followed by sonication five times on ice at 60% pulse for 30 sec with 30 sec intervals. Bacterial debris was removed by centrifugation at 8,000×g at 4 °C for 20 min. The supernatant containing soluble 6×His tagged protein was subjected to the affinity chromatography column packed with glutathione Ni- sepharose beads (GE Healthcare) and pre-equilibrated with lysis buffer (without lysozyme) at 4 °C. After 1 h incubation, the column was washed with five column volumes pre-chilled wash-buffer (Tris 50 mM, NaCl 150 mM, β-Mercaptoethanol 0.1%). The column-bound 6×His tagged- antibody was recovered from the column using elution buffer containing imidazole 500 mM, Na H_2_PO_4_ pH 7.4, and NaCl 500 mM. Finally, the purified proteins were dialyzed against Tris 50 mM pH 8, NaCl 150 mM buffer, and analyzed on SDS-PAGE to monitor the protein production and purification. Western blotting technique was used to verify the expression of antibodies in which mouse anti-His primary antibody (1:3000 diluted in BSA) and goat anti-mouse IgG-HRP secondary antibody (1:8000 diluted in BSA) were used for protein detection. The protein bands were visualized by applying enhanced chemiluminescence (ECL) reagent.

*ELISA experiment*

The functionality of the produced dAbs was tested by ELISA experiment. To this end, TNF-α (100 µg/mL) prepared in house as described previously in (24), was coated on a 96-well plate. After 2 h blocking using 3% BSA at room temperature, different concentrations of each purified dAb were prepared in TBS buffer (10 mM Tris–HCl, 100 mM NaCl, pH 8.0) and added to the TNF-α coated wells with incubation for 1.5 h at room temperature with gentle shaking. Following the incubation, the wells were washed four times with TBST (TBS with 0.05% tween 20). Subsequently, 100 µL of anti-6×His antibody at 1:1000 diluted in 2% BSA was added to each well, and the plate was incubated for 1 h at room temperature with gentle shaking. After washing four times with TBST, the wells were treated with 100 µL of 1:3000 diluted HRP-conjugated goat anti mouse antibody for 1 h. Finally, 100 µL substrate solution containing TMB 100 µg/mL, prepared in potassium acetate (100 mM, pH 6.0) and hydrogen peroxide (0.006% v/v) was added to each well. The enzymatic reaction was terminated after 15 min using 50 µL of 1M H_2_SO_4_. The absorbance was measured at 450 nm and 650 nm using ELISA plate reader. TNF-α uncoated wells and TNF-α coated wells without addition of dAb were used as the controls. All data points were the average of three individual experiments. The results were fit into one-site specific binding curve using Prism program (version 6.01, GraphPad Software Inc.) in order to calculate the K_d_ values.

*MTT assay*

Murine L929 fibroblast cells were cultured in RPMI supplemented with 10% FBS. The cells were harvested in log phase growth using trypsin-EDTA. L929 cell suspension including approximately 10,000 cells were seeded in each well of 96-well plate and incubated for 24 h at 37 °C under a humidified atmosphere and 5% CO_2_. Different concentrations of the affinity purified dAbs (ranging from 0.023 to 1.83 µM for I44 and 0.047 to 3.82 µM for I49) were added to 0.0057 nM of TNF-α and the mixtures were incubated for 24 h in the presence of Actionomycin (1 µg/mL) at 37 °C. The mixtures were then added to the seeded cells and incubated for additional 24 h. Adalimumab, a marketed anti-TNF-α antibody at different concentration between 6.76E-5 and 6.76 nM, were used as the positive control. After incubation, 20 µL of MTT (5 mg/mL) was added to each well and the plate was kept in the incubator for 4 h. After discarding the supernatant, 100 µL of solublizing buffer containing Sorensen buffer (12.5%) and DMSO (87.5%) was added to each well and incubated for 40 min at room temperature with agitation. Finally, the absorbance was measured at 570 nm. For each concentration the experiment was carried out in triplicate wells. The achieved results represented as the percentages of TNF-α neutralizing effects in comparison to the TNF-α treated (maximum toxicity) and untreated cells (maximum viability) were fit into dose-response inhibition curve using Prism program (version 6.01, GraphPad Software Inc.).

*Molecular modeling and docking study*

**Figure 1:**
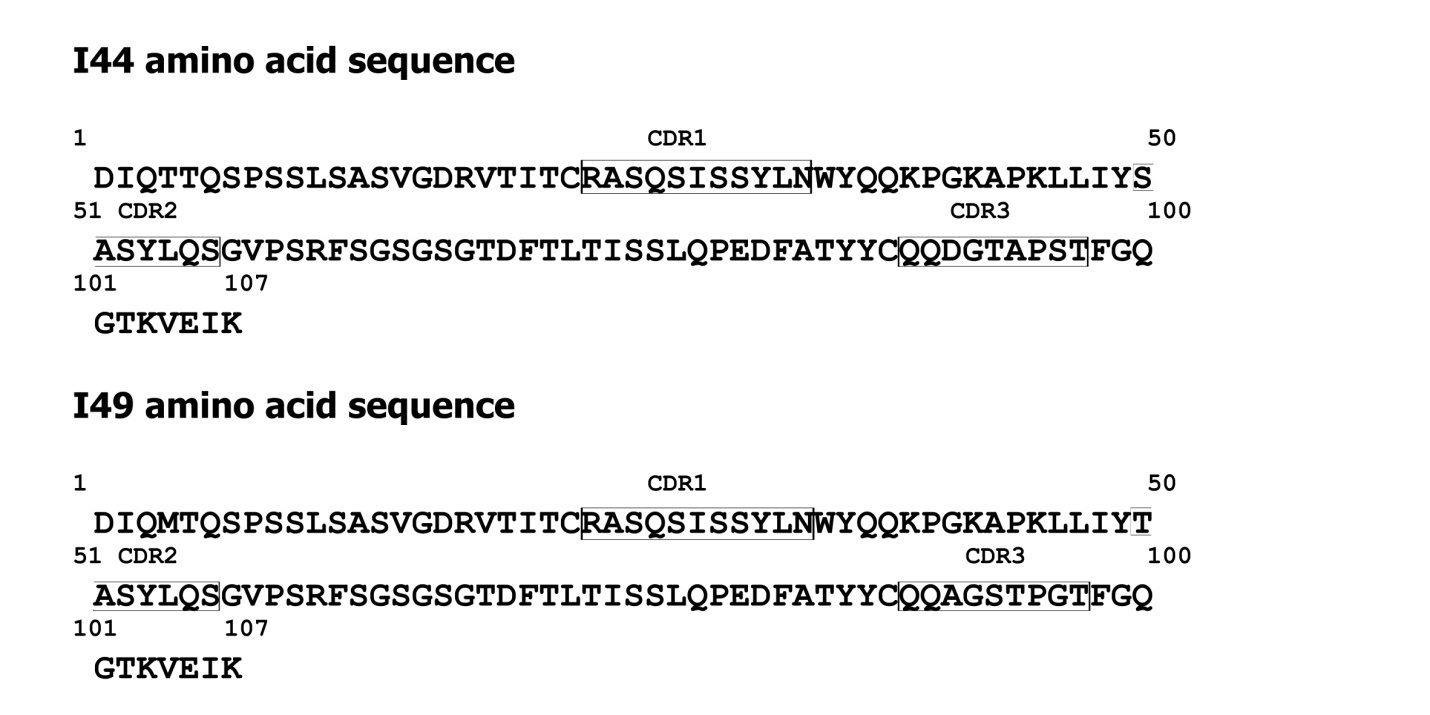
Amino acid sequences of I44 and I49 dAbs. The CDRs have been shown on the sequences

The crystal structure of TNF-α trimer was retrieved from protein data bank (PDB ID: 1TNF). In order to construct the 3D-structure of I44 and I49 dAbs from the corresponding amino acid sequences (Figure 1), the homology modeling was utilized using Swiss Model web server (25-28). The appropriate template was selected based on QMEAN value. The quality and geometrical features of the generated models were checked using PROCHECK, MolProbity, and Verify-3D programs (29-31). The ZDOCK program, running under Fedora/CentOS LINUX operating system with two dual-core Opteron 2212 CPUs and 2GB RAM, was employed to dock the modeled dAbs onto the TNF-α trimer. The protein interaction calculator (PIC) web server was used to extract the possible interactions between the TNF-α and the identified anti-TNF-α antibodies (32).

## Results

*Expression and purification of anti –TNF-α domain antibodies*

**Figure 2:**
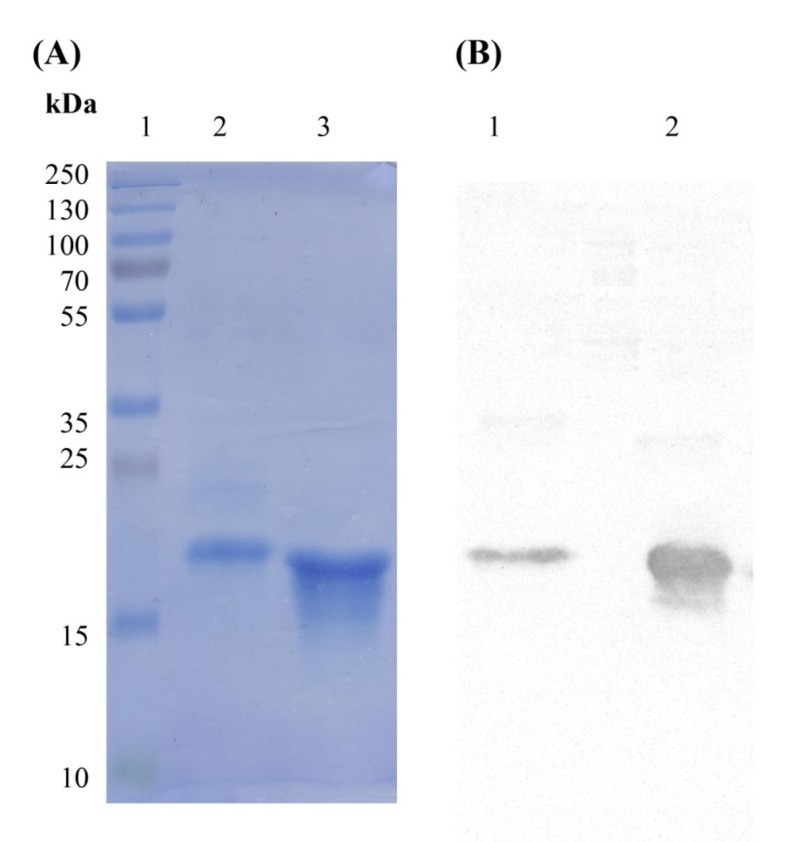
SDS-PAGE and western blotting analyses of I44 and I49 domain antibodies. The band around 17 kDa represents the produced I44 and I49 domain antibodies. (A) lane 1 is the protein weight marker, lane 2 represents the purified I44 and lane 3 is the purified sample of I49. (B) The western blot analysis of the samples shown in A. Lanes 2 and 3 correspond to lanes 2 and 3 in A

Phage display technology is a powerful tool for isolating specific peptide/antibody binders against target of interest. In this method, the coding genes of various peptides/antibodies are cloned into genome of phage in order to be displayed on one of the phage coat proteins. Therefore, a library of phage each representing an individual peptide/antibody is generated which can be screened against any target. In our previous study, two phage displaying dAbs capable of binding to TNF-α were identified using phage display technology (23). The current investigation was intended to produce and purify these dAbs for further evaluation of their binding characteristics to TNF-α. The coding sequences of I44 and I49 dAbs in pIT2 phage vector are followed by 6×His tag coding segment, enabling His tagged expression of these antibodies as fusion proteins. For expression, phage displaying dAbs were infected into *E. coli origami* *(DE3) *expression system induced by 1mM IPTG under temperature of 30 °C for overnight. The produced I44 and I49 antibodies were purified on affinity column resulting in 0.28 and 0.6 mg purified proteins, respectively (Table 1). Analyzing the samples from different steps of protein production by SDS-PAGE, indicated successful expression and purification of proteins with distinguished bands at about 17 kDa (Figure 2A). Further confirmation for protein expression was carried out using western blotting technique in which the corresponding protein bands (around 17 kDa) were detected using ECL (Figure 2B). 

*Evaluation of binding ability of I44 and I49 domain antibodies to TNF-α*

**Figure 3:**
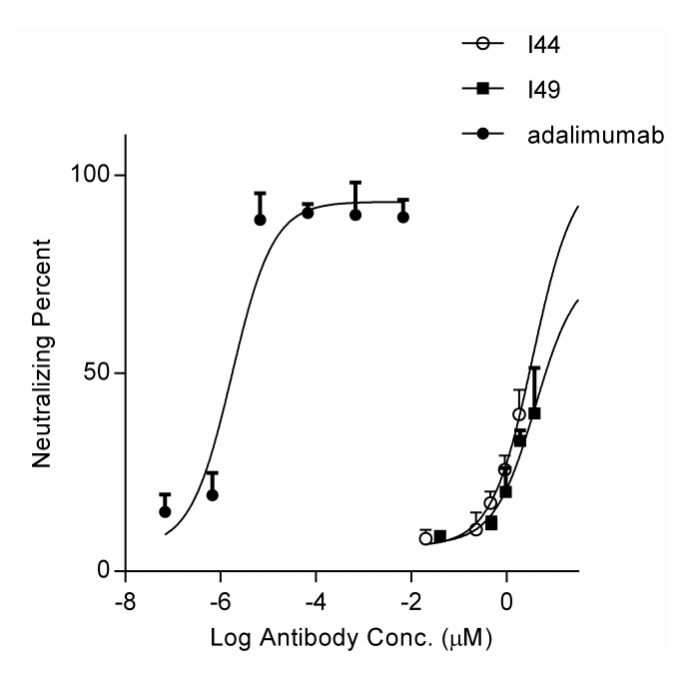
ELISA experiment using different concentrations of I44 and I49 domainantibodies. Various concentrations of dAbs were added to the TNF-α coated wells. Subsequently, mouse anti-6×His and goat anti-mouse HRP-conjugated antibodies were used for protein detection. All data are the means of triplicate ± SD

For determination of TNF-α binding capability of the identified dAbs, ELISA experiment, was conducted. The purified dAbs (*i.e.* I44 and I49) in different concentrations ranging from 0.35 to 5.83 µM were added to 96-well plate coated with TNF-α. Then, anti-6×His and HRP-conjugated goat anti-mouse antibodies were sequentially used to evaluate the TNF-α binding ability of dAbs. Analyzing of the results showed that I44 and I49 dAbs bind to TNF-α with K_d_ values of 5.18 ± 1.41 and 2.42 ± 0.55 µM, respectively (Figure 3). The obtained data from Prism analysis have been summarized in Table 2.

**Figure 4:**
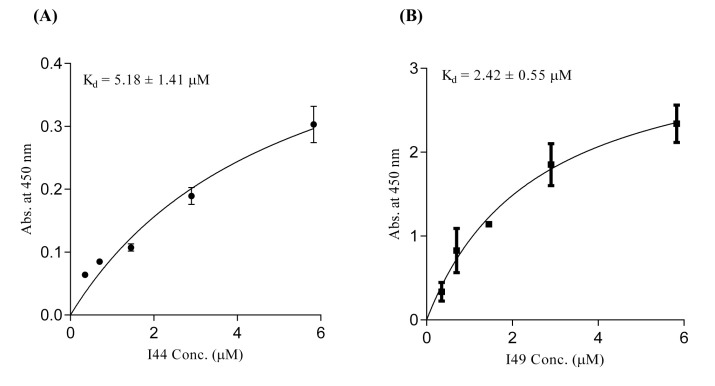
MTT assay using the purified I44 and I49 dAbs as the inhibitors of TNF-α cytotoxic effect. Different concentrations of dAbs (ranging from 0.023 to 1.83 µM for I44 and 0.047 to 3.82 µM for I49) and adalimumab was incubated with 0.0057 nM of TNF-α before addition onto L929 cells. After 24 h treatment, the neutralizing potency of I44 and I49 dAbs on TNF-α induced cell cytotoxicity was determined. Each data point is the average of three independent experiments, and the error bar is the corresponding standard error calculated by Prism program

**Figure 5:**
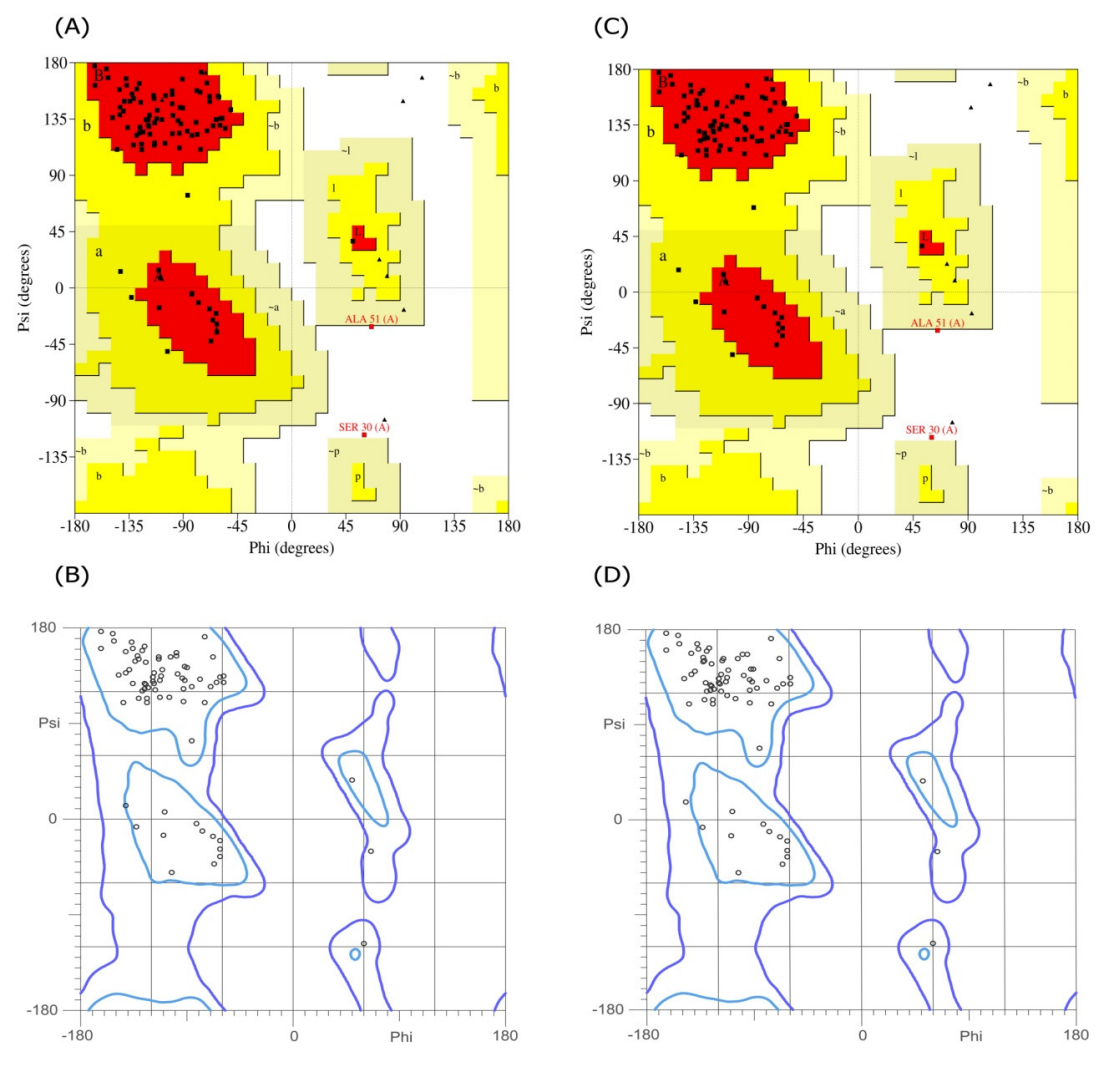
The Ramachandran plots for the I44 and I49 models. The (A) and (B) are the plots obtained for I44 dAb using PROCHECK and Molprobity web servers, respectively, while the same plots for I49 are shown in (C) and (D). Both calculations showed that more than 97.7% of amino acids are in the allowed regions

**Figure 6:**
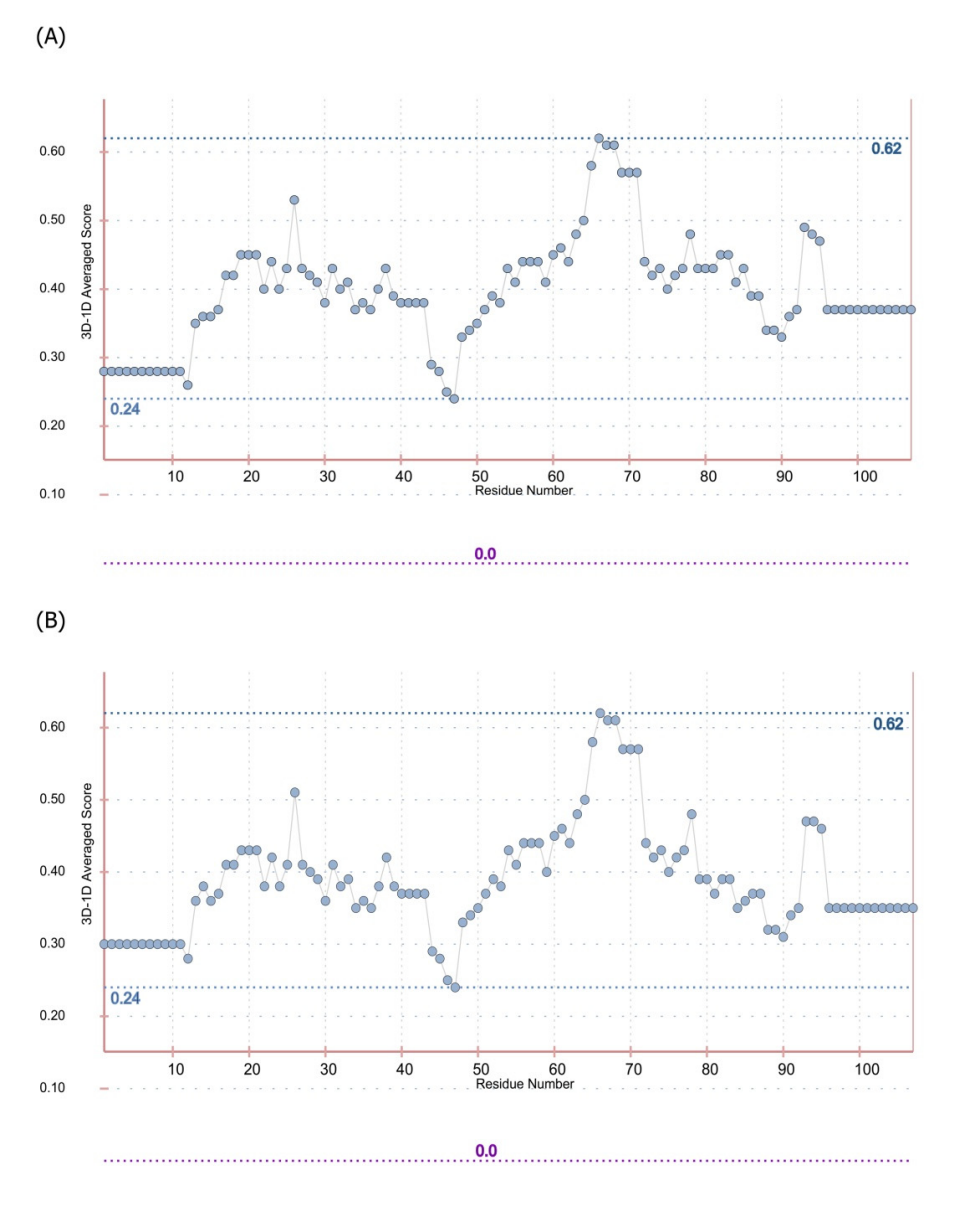
The results of sequence-structure compatibility evaluation for I44 (A) and I49 (B) models. The results are based on Profiles- 3D method calculated by Verify-3D web server. The positive scores suggest that the residues are placed in a proper environment and the protein is folded correctly

**Figure 7:**
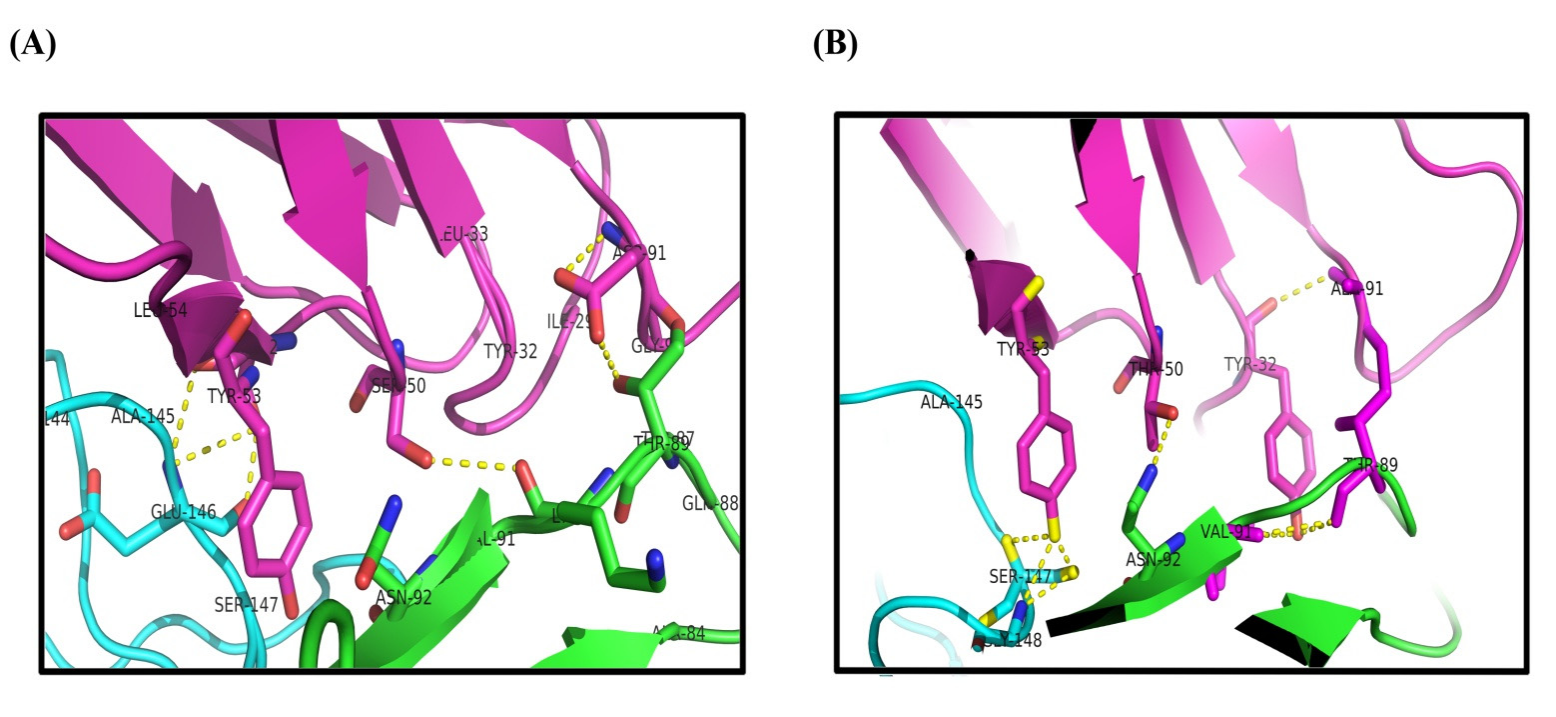
Cartoon representation of I44- TNF-α (A) and I49-TNF-α (B) complexes. The residues involved in hydrogen bonds have been shown as stick representation generated in PyMOL (version 1.5.0.3)

**Table 1:** Purification of I44 and I49 dAbs from 500 mL culture of E. coli origami infected with phage displaying I44 and I49 dAbs. Protein concentration was measured using Bradford method

	**Culture volume**	**Bacterial mass**	**Amount of purified protein**
I44	500 mL	4.16 g	0.28 mg
I49	500 mL	3.16 g	0.60 mg

**Table 2:** Determination of the dissociation constants for the selected domain antibodies toward TNF-α assessed using ELISA experiment

**Antibodies**	**B** **max ** **(AU at 450 nm)**	**K** **d ** **(µM)**		
	**Mean**	**SE**	**Mean**	**SE**
I44	0.56	0.09	5.18	1.41
I49	3.39	0.33	2.57	0.55

**Table 3:** List of possible interactions between TNF-α and dAbs predicted by PIC web server. TNF-α residues have been numbered according to residue numbers in TNF-α crystal structure retrieved from protein data bank (1TNF) and dAbs residue numbers follow the rule in Figure 1

**Interaction type**	**TNF-α residue number**	**I44 residue number**	**I49 residue number**
Hydrophobic contacts	Ala84	Tyr32	-
	Tyr87	Ile29, Tyr32	Ile29, Tyr32
	Val91	Tyr32	Tyr32
	Leu143	Leu54	Leu54
	Phe144	Leu54	-
	Ala145	Tyr53, Leu54	Tyr53, Leu54
Hydrogen bonds	Gln88	Gly92	-
	Glu146	Ser52	-
	Ala145	Tyr53	-
	Tyr87	Ile29, Tyr32	Ile29
	Thr89	Asp91	Ala91
	Asn92	Ser50	Thr50
	Glu146	Ser52	-
	Ser147	Tyr53	Tyr53
	Gly148	Tyr53	Tyr32
	Thr89	Tyr32	Tyr32
	Thr87	Leu33	Ser30, Ser31, Tyr32
	Lys90	Ser50	-
	Asn92	Tyr53	Thr50
	Thr89	Gly92, Asp91	-
	Val91	-	Tyr32

*Inhibition of TNF-α cytotoxicity by domain antibodies*

The MTT assay was performed to evaluate the ability of the selected dAbs to inhibit TNF-α cytotoxicity on L929 cells. Pre-incubation of TNF-α with these dAbs at the different concentrations revealed that I44 and I49 antibodies could inhibit TNF-α cytotoxicity with IC_50_ values of 6.61 ± 2.32 and 3.64 ± 1.89 µM, respectively. Adalimumab, used as the positive control, prevented TNF-α induced cytotoxicity with IC_50_ value of 1.67 ± 0.32 pM (Figure 4).

*Investigation of the interaction modes between TNF-α and domain antibodies*

In order to predict the interaction modes between the identified dAbs and TNF-α, the structures of dAbs were modeled using Swiss Model web server. The quality of the models was evaluated using PROCHECK and MolProbity programs (Figure 5). Analyzing the results showed that more than 97.7% of residues are in the allowed regions indicative of the high quality of the model from geometrical viewpoint. Further model validation was carried out using Verify-3D methodology, in which the compatibility of the model sequence within the proposed 3D structure was assessed (30, 33). Data analysis showed that the 3D–1D scores for 95.04% (more than 80%) of the residues were more than 0.2 and hence, the quality criteria defined by the program was passed by the model (Figure 6). In the next step, molecular docking study was performed and the built models for dAbs were docked onto TNF-α trimer using ZDOCK program (Figure 7). Using PIC web server the possible interactions between the dAbs and TNF-α were investigated and the results are shown in Table 3.

## Discussion

Progress of inflammatory conditions greatly depends on TNF-α aberrant production. Therefore, prevention of TNF-α activity would result in promising outcomes in patients suffering from inflammatory diseases. In this regard much attention has been devoted to develop anti-TNF-α agents which could directly bind to TNF-α suppressing its adverse effects. These inhibitory molecules vary from small molecules such as peptides to macromolecules such as full length antibodies (34-42). Up to now, only few full length anti-TNF-α antibodies have been approved by FDA to be used in inflammatory diseases (12). Antibodies are biological agents that are extensively used in clinic and diagnosis due to their high specificity and affinity towards corresponding antigens (20). Despite having the advantage of specificity, the immunogenicity and pharmacokinetic problems as well as the time consuming and expensive process of production of such therapeutic antibodies are challenging issues (43-48). Therefore, identification of small format of antibodies which provide manageable physicochemical properties with suitable specificity towards target of interest is of great importance. Such attitude paved the way for introducing the therapeutic Fab antibodies such as abciximab (an anti-PIIb/IIIa), ranibizumab (an anti-VEGF), and certolizumab pegol (an anti-TNF-α) (49-51). Tomlinson I&J phage display antibody libraries are non-immunized human phage libraries composed of scFv displaying phagemids developed by Greg Winter and colleagues in 2000 (52). Although Tomlinson phage libraries are designed based on scFv format, in our panning process against TNF-α, two antibodies namely, I44 and I49, possessing only one domain of scFv antibodies (*i.e.* V_L_) were identified (23). Compared to larger formats of antibodies such as full length, Fab, and scFv antibodies, domain antibodies (dAbs) are more stable non-aggregating molecules, which make them suitable tools for many purposes such as inhibition of cytosolic/nuclear proteins that cannot be targeted with genetic knockout techniques due to the much easier folding of dAbs inside the cytosol (53). On the other hand, because of the small size, domain antibodies can be administered not only by injection but also from oral route. These excellent features of dAbs compared to complete antibodies and scFv fragments have attracted much interest of different companies around the world such as Ablynx, Domantis (GSK), Ossianix to investigate on identification of new dAbs for therapeutic purposes. Ozoralizumab, produced by Ablynx company for rheumatoid arthritis, is anti-TNF-α single domain antibody which has successfully passed phase IIa clinical trial study. Furthermore, several therapeutic domain antibodies are in different phases of preclinical and clinical trials in Ablynx, Domantis (GSK), and Ossianix companies. For example, caplacizumab (ALX-0081) is the first single domain anti-von Willebrand factor antibody which is going to be marketed for treatment of thrombotic thrombocytopenic purpura and thrombosis by Ablynx Company (53). Based on this evidence, we aimed to produce and purify the previously identified single domain anti-TNF-α antibodies namely, I44 and I49, in order to investigate their binding to and inhibition of TNF-α. To do so, phage particles harboring the coding gene for I44 and I49 were used to infect *E.coli origami (DE3)* bacteria. Presence of PelB signal peptide in phage genome enables periplasmic expression of the target proteins (*i.e.*, I44 and I49). This feature provides an advantage for the current expression system where periplasmic compartment supplies a reduced environment suitable for the formation of disulfide bonds in 3D structures of I44 and I49 proteins (54, 55). This expression and purification system resulted in successful production of I44 and I49 antibodies with high purity, evidenced by SDS-PAGE and western blotting analyses (Figure 2). The variation in the amount of produced I44 and I49 proteins is understandable as few differences in amino acid sequences of proteins can result in much variability in protein production in the same condition (56). In ELISA experiment I44 and I49 showed appropriate affinity towards TNF-α with K_d_ values of 5.18 ± 1.41 and 2.42 ± 0.55 µM, respectively, which are comparable with results reported elsewhere for small format anti-TNF-α antibodies such as scFvs (57, 58). The inhibitory effect of I44 and I49 antibodies on TNF-α biological activity was evaluated by MTT assay where TNF-α sensitive L929 cells were survived from TNF-α cytotoxic effects in the presence of different concentrations of the dAbs. The determined IC_50_ for I44 and I49 were 6.61 and 3.64 µM, respectively which are much bigger than that of adalimumab (1. 67 ± 0.32 pM). Such big difference in IC_50_ values can be attributed to the lower binding valence of I44 and I49 single dAbs (~17 kDa) compared to adalimumab (148 kDa). To identify the most important residues involved in antibodies-TNF-α interactions, molecular docking was carried out in which the modeled structures of dAbs were docked onto TNF-α trimer. The docking scores obtained from ZDock program were 952.23 and 981.76 for I44-TNF-α and I49-TNF-α complexes, respectively, which is in close agreement with the data achieved from ELISA experiment where I49 showed higher affinity towards TNF-α compared to I44. In the next step, the possible interactions between dAbs and TNF-α were predicted using PIC web server (Table 3). According to PIC analyses, the main hydrophobic contacts within 5 Å between dAbs and TNF-α are established via Tyr^32^ in CDR1 of both antibodies and Tyr^87^ and Val ^91^ of TNF-α (Figure 7). In addition, Tyr^53^ and Leu^54^ located at CDR2 of dAbs are also contributing in hydrophobic interactions involving Leu^143^ and Ala^145^ of TNF-α (Figure 7). These results are in close agreement with the crystallographic data of adalimumab-TNF-α complex where the hydrophobic interaction of adalimumab with Val^91^ of TNF-α has been elucidated (59). Apart from hydrophobic contacts, dAbs-TNF-α complexes have been stabilized by hydrogen bonds. The most important hydrogen bonds are detected between I44 Ser^50^ and I49 Thr^50^ at CDR2 of antibodies and Asn^92^ of TNF-α as well as I44 Tyr^32^ (CDR1) and I49 Tyr^53^ (CDR2) with Val^91^ and Gly^148^ of TNF-α, respectively (Figure 7). Such intermolecular hydrogen bonds are observed in the crystal structure of adalimumab-TNF-α complex (59). Comparison of the possible interactions of these dAbs with the interactions of adalimumab and infliximab with TNF-α revealed that the interaction mode of I44 and I49 is similar to adalimumab rather than infliximab. It has been shown that four distinguished clusters of TNF-α consisting of residues ranging from 20-23, 65-92, 110-115, and 135-146, are participating in adalimumab binding (59). Comparing this information with the data obtained from molecular docking study indicates that the identified dAbs mimic the binding mode of adalimumab, interacting with TNF-α residues ranging from 84-92 and 143-148. However, no interactions between the dAbs and TNF-α residues ranging from 20-23 and 110-115 were observed which can be reasoned for their low effectiveness compared to adalimumab. Therefore, structural modifications on the identified dAbs for designing more potent anti-TNF-α antibodies are required. This goal can be achieved by altering the key residues of the antibodies responsible for TNF-α binding in order to determine the role of each residue in TNF-α binding to propose mutations for improvement of TNF-α binding characteristic of the designed antibodies. Moreover, TNF-α binding abilities of the dAbs, identified in the current study, can be refined by pairing these antibodies with a library of heavy chain phage libraries (chain-shuffling) (60, 61). 

## Conclusion

In the current study, the previously identified anti-TNF-α dAbs, I44 and I49, were recombinantly produced and purified. The binding properties of the prepared antibodies were determined using experimental and molecular modeling techniques. Based on experimental assays, these dAbs are capable of binding to and inhibiting TNF-α cytotoxic effects. Molecular docking studies identified the key residues important in the interactions between the studied domain antibodies and TNF-α. The information obtained in the current study can be useful for further structural studies and designing potent anti-TNF-α small format antibodies.

## References

[1] Esposito E, Cuzzocrea S (2009). TNF-alpha as a therapeutic target in inflammatory diseases, ischemia-reperfusion injury and trauma. Curr. Med. Chem.

[2] Idriss HT, Naismith JH (2000). TNFα and the TNF receptor superfamily: Structure-function relationship (s). Microsc. Res. Tech.

[3] Carswell EA, Old LJ, Kassel RL, Green S, Fiore N, Williamson B (1975). An endotoxin induced serum factor that cuases necrosis of tumors. Proc. Natl. Acad. Sci. U. S. A.

[4] Moss ML, Sklair-Tavron L, Nudelman R (2008). Drug Insight: Tumor necrosis factor-converting enzyme as a pharmaceutical target for rheumatoid arthritis. Nat. Clin. Pract. Rheumatol.

[5] Scheiermann C, Kunisaki Y, Frenette PS (2013). Circadian control of the immune system. Nat. Rev. Immunol.

[6] Keller M, Mazuch J, Abraham U, Eom GD, Herzog ED, Volk HD, Kramer A, Maier B (2009). A circadian clock in macrophages controls inflammatory immune responses. Proc. Natl. Acad. Sci. U. S. A.

[7] Cleynen I, Vermeire S (2012). Paradoxical inflammation induced by anti-TNF agents in patients with IBD. Nature Reviews Gastroenterology and Hepatology.

[8] Ricart E, Ordás I, Panés J (2012). Anti-TNF antibody therapy in Crohn′s disease: The risk of a switch. Gut.

[9] Brietzke E, Kapczinski F (2008). TNF-α as a molecular target in bipolar disorder. Prog. Neuropsychopharmacol. Biol. Psychiatry.

[10] Monaco C, Nanchahal J, Taylor P, Feldmann M (2015). Anti-TNF therapy: Past, present and future. Int. Immunol.

[11] Thalayasingam N, Isaacs JD (2011). Anti-TNF therapy. Best Practice and Research: Clin. Rheumatol.

[12] Jarrot PA, Kaplanski G (2014). Anti-TNF-alpha therapy and systemic vasculitis. Mediators Inflamm.

[13] Elliott MJ, Maini RN, Feldmann M, Kalden JR, Antoni C, Smolen JS, Leeb B, Breedveld FC, Macfarlane JD, Bijl JA, Woody JN (1994). Randomised double-blind comparison of chimeric monoclonal antibody to tumour necrosis factor α (cA2) versus placebo in rheumatoid arthritis. Lancet.

[14] Palladino MA, Bahjat FR, Theodorakis EA, Moldawer LL (2003). Anti-TNF-α therapies: The next generation. Nat. Rev. Drug Discov..

[15] Sandborn WJ, Hanauer SB, Katz S, Safdi M, Wolf DG, Baerg RD, Tremaine WJ, Johnson T, Diehl NN, Zinsmeister AR (2001). Etanercept for active Crohn′s disease: A randomized, double-blind, placebo-controlled trial. Gastroenterology.

[16] Tracey D, Klareskog L, Sasso EH, Salfeld JG, Tak PP (2008). Tumor necrosis factor antagonist mechanisms of action: A comprehensive review. Pharmacol. Ther.

[17] Gómez-Reino JJ, Carmona L, Rodríguez Valverde V, Mola EM, Montero MD (2003). Treatment of rheumatoid arthritis with tumor necrosis factor inhibitors may predispose to significant increase in tuberculosis risk: A multicenter active-surveillance report. Arthritis Rheumatol.

[18] Lubel JS, Testro AG, Angus PW (2007). Hepatitis B virus reactivation following immunosuppressive therapy: Guidelines for prevention and management. Intern. Med. J.

[19] Nyboe Andersen N, Pasternak B, Friis-Møller N, Andersson M, Jess T (2015). Association between tumour necrosis factor-α inhibitors and risk of serious infections in people with inflammatory bowel disease: nationwide Danish cohort study. BMJ (Clinical research ed.).

[20] Ahmad ZA, Yeap SK, Ali AM, Ho WY, Alitheen NBM, Hamid M (2012). ScFv antibody: Principles and clinical application. Clin. Dev. Immunol.

[21] Jain S, Aresu L, Comazzi S, Shi J, Worrall E, Clayton J, Humphries W, Hemmington S, Davis P, Murray E, Limeneh AA, Ball K, Ruckova E, Muller P, Vojtesek B, Fahraeus R, Argyle D, Hupp TR (2016). The development of a recombinant scFv monoclonal antibody targeting canine CD20 for use in comparative medicine. PLoS ONE.

[22] Nelson AL (2010). Antibody fragments: Hope and hype. MAbs.

[23] Alizadeh AA, Hamzeh-Mivehroud M, Dastmalchi S (2015). Identification of novel single chain fragment variable antibodies against tnf-α using phage display technology. Adv. Pharm. Bull..

[24] Alizadeh AA, Hamzeh-Mivehroud M, Farajzadeh M, Moosavi-Movahedi AA, Dastmalchi S (2015). A simple and rapid method for expression and purification of functional TNF-α using GST fusion system. Curr. Pharm. Biotechnol.

[25] Guex N, Peitsch MC, Schwede T (2009). Automated comparative protein structure modeling with SWISS-MODEL and Swiss-PdbViewer: a historical perspective. Electrophoresis.

[26] Kiefer F, Arnold K, Künzli M, Bordoli L, Schwede T (2009). The SWISS-MODEL Repository and associated resources. Nucleic Acids Res.

[27] Arnold K, Bordoli L, Kopp J, Schwede T (2006). The SWISS-MODEL workspace: a web-based environment for protein structure homology modelling. Bioinformatics.

[28] Biasini M, Bienert S, Waterhouse A, Arnold K, Studer G, Schmidt T, Kiefer F, Gallo Cassarino T, Bertoni M, Bordoli L, Schwede T (2014). SWISS-MODEL: modelling protein tertiary and quaternary structure using evolutionary information. Nucleic Acids Res.

[29] Weiss MS, Anderson DH, Raffioni S, Bradshaw RA, Ortenzi C, Luporini P, Eisenberg D (1995). A cooperative model for receptor recognition and cell adhesion: Evidence from the molecular packing in the 16-Å crystal structure of the pheromone Er-1 from the ciliated protozoan Euplotes raikovi. Proc. Natl. Acad. Sci. U. S. A.

[30] Luthy R, Bowie JU, Eisenberg D (1992). Assessment of protein models with three-dimensional profiles. Nature.

[31] Laskowski RA, Rullmann JAC, MacArthur MW, Kaptein R, Thornton JM (1996). AQUA and PROCHECK-NMR: Programs for checking the quality of protein structures solved by NMR. J. Biomol. NMR.

[32] Tina K, Bhadra R, Srinivasan N (2007). PIC: protein interactions calculator. Nucleic acids Res.

[33] Bowie JU, Luthy R, Eisenberg D (1991). A method to identify protein sequences that fold into a known three-dimensional structure. Science.

[34] Alizadeh AA, Hamzeh-Mivehroud M, Farajzadeh M, Dastmalchi S (2017). Identification of novel peptides against TNF-α using phage display technique and in silico modeling of their modes of binding. Eur. J. Pharm. Sci.

[35] Brunetti J, Lelli B, Scali S, Falciani C, Bracci L, Pini A (2014). A novel phage-library-selected peptide inhibits human TNF-alpha binding to its receptors. Molecules.

[36] Chirinos-Rojas CL, Steward MW, Partidos CD (1997). Use of a solid-phase random peptide library to identify inhibitors of TNF-alpha mediated cytotoxicity in-vitro. Cytokine.

[37] Chirinos-Rojas CL, Steward MW, Partidos CD (1998). A peptidomimetic antagonist of TNF-alpha-mediated cytotoxicity identified from a phage-displayed random peptide library. J. Immunol.

[38] Chirinos-Rojas CL, Steward MW, Partidos CD (1999). A phage-displayed mimotope inhibits tumour necrosis factor-alpha-induced cytotoxicity more effectively than the free mimotope. Immunology.

[39] Guo HP, Luo HB, Liu YJ, Zhu P, Fu N (2002). Screening of tumor necrosis factor-alpha-binding peptides by phage display peptide library. Di Yi Jun Yi Da Xue Xue Bao.

[40] Sclavons C, Burtea C, Boutry S, Laurent S, Vander Elst L, Muller RN (2013). Phage display screening for tumor necrosis factor- alpha -binding peptides: detection of inflammation in a mouse model of hepatitis. Int. J. Pept..

[41] Zhang J, Zheng L, Zhao A, Gao B, Liu NL, Wang F, Dong J, Xin ZT, Shao NS, Wang HX, Xue YN (2003). Identification of anti-TNFα peptides with consensus sequence. Biochem. Biophys. Res. Commun..

[42] Kalliolias GD, Ivashkiv LB (2016). TNF biology, pathogenic mechanisms and emerging therapeutic strategies. Nat. Rev. Rheumatol.

[43] Antoni C, Braun J (2002). Side effects of anti-TNF therapy: current knowledge. Clin. Exp. Rheumatol.

[44] Debandt M, Vittecoq O, Descamps V, Le Loet X, Meyer O (2003). Anti-TNF-alpha-induced systemic lupus syndrome. Clin. Rheumatol.

[45] Devos SA, Van Den Bossche N, De Vos M, Naeyaert JM (2003). Adverse skin reactions to anti-TNF-alpha monoclonal antibody therapy. Dermatology.

[46] Dias OM, Pereira DA, Baldi BG, Costa AN, Athanazio RA, Kairalla RA, Carvalho CR (2014). Adalimumab-induced acute interstitial lung disease in a patient with rheumatoid arthritis. J. Bras. Pneumol.

[47] Shanahan F (2000). Anti-TNF therapy for Crohn′s disease: a perspective (infliximab is not the drug we have been waiting for). Inflamm. Bowel. Dis.

[48] Wolf R, Matz H, Orion E, Ruocco V (2002). Anti-TNF therapies--the hope of tomorrow. Clin. Dermatol.

[49] Investigators E (1994). Use of a monoclonal antibody directed against the platelet glycoprotein IIb/IIIa receptor in high-risk coronary angioplasty. N. Engl. J. Med.

[50] Nixon AE, Sexton DJ, Ladner RC (2014). Drugs derived from phage display: from candidate identification to clinical practice. MAbs.

[51] Sandborn WJ (2008). Certolizumab pegol for moderate-to-severe Crohn′s disease. Gastroenterol. Hepatol.

[52] de Wildt RM, Mundy CR, Gorick BD, Tomlinson IM (2000). Antibody arrays for high-throughput screening of antibody–antigen interactions. Nat. Biotechnol.

[53] Boldicke T (2017). Single domain antibodies for the knockdown of cytosolic and nuclear proteins. Protein Sci.

[54] de Marco A (2009). Strategies for successful recombinant expression of disulfide bond-dependent proteins in Escherichia coli. Microb. Cell Fact.

[55] Kipriyanov SM, Aitken R (2009). High-Level Periplasmic Expression and Purificationof scFvs. Antibody Phage Display: Methods and Protocols.

[56] Miethe S, Meyer T, Wöhl-Bruhn S, Frenzel A, Schirrmann T, Dübel S, Hust M (2013). Production of single chain fragment variable (scFv) antibodies in Escherichia coli using the LEX™ bioreactor. J. Biotechnol.

[57] Chang H, Qin W, Li Y, Zhang J, Lin Z, Lv M, Sun Y, Feng J, Shen B (2007). A novel human scFv fragment against TNF-α from de novo design method. Mol. Immunol.

[58] Geng S, Chang H, Qin W, Lv M, Li Y, Feng J, Shen B (2015). A novel anti-TNF scFv constructed with human antibody frameworks and antagonistic peptides. Immunol. Res.

[59] Hu S, Liang S, Guo H, Zhang D, Li H, Wang X, Yang W, Qian W, Hou S, Wang H (2013). Comparison of the inhibition mechanisms of adalimumab and infliximab in treating tumor necrosis factor α-associated diseases from a molecular view. J. Biol. Chem.

[60] Mukamoto M, Maeda H, Kohda T, Nozaki C, Takahashi M, Kozaki S (2012). Characterization of neutralizing mouse-human chimeric and shuffling antibodies against botulinum neurotoxin A. Microbiol Immunol.

[61] Nishi M, Jian N, Yamamoto K, Seto H, Nishida Y, Tonoyama Y, Shimizu N, Nishi Y (2014). Ligation-based assembly for constructing mouse synthetic scFv libraries by chain shuffling with in-vivo-amplified VH and VL fragments. J. Immunol. Methods.

